# Markers of Tissue-Specific Insulin Resistance Predict the Worsening of Hyperglycemia, Incident Type 2 Diabetes and Cardiovascular Disease

**DOI:** 10.1371/journal.pone.0109772

**Published:** 2014-10-13

**Authors:** Mária FízeI'ová, Henna Cederberg, Alena Stančáková, Raimo Jauhiainen, Jagadish Vangipurapu, Johanna Kuusisto, Markku Laakso

**Affiliations:** 1 Department of Medicine, University of Eastern Finland, Kuopio, Finland; 2 Department of Medicine, University of Eastern Finland and Kuopio University Hospital, Kuopio, Finland; Azienda Ospedaliero-Universitaria Careggi, Italy

## Abstract

We investigated the ability of surrogate markers of tissue-specific insulin resistance (IR, Matsuda IR, Adipocyte IR, Liver IR) to predict deterioration of hyperglycemia, incident type 2 diabetes and cardiovascular events in the Metabolic Syndrome in Men (METSIM) Study. The METSIM Study includes 10,197 Finnish men, aged 45–73 years, and examined in 2005–2010. A total of 558 of 8,749 non-diabetic participants at baseline were diagnosed with new-onset diabetes and 239 with a new CVD event during a 5.9-year follow-up of this cohort (2010–2013). Compared to fasting plasma insulin level, Matsuda IR (IR in skeletal muscle) and Adipocyte IR were significantly better predictors of 2-hour plasma glucose and glucose area under the curve after adjustment for confounding factors. Liver IR was the strongest predictor of both incident type 2 diabetes (hazard ratio = 1.83, 95% confidence interval: 1.68–1.98) and cardiovascular events (hazard ratio = 1.31, 95% confidence interval: 1.15–1.48). Hazard ratios for fasting insulin were 1.37 (95% confidence interval: 1.32–1.42) and 1.11 (95% confidence interval: 1.00–1.24), respectively. Tissue-specific markers of IR, Matsuda IR and Adipocyte IR, were superior to fasting plasma insulin level in predicting worsening of hyperglycemia, and Liver IR was superior to fasting insulin level in predicting incident type 2 diabetes and cardiovascular events.

## Introduction

Insulin resistance (IR) and impaired insulin secretion are two major pathophysiological defects in type 2 diabetes [1]. Insulin resistance, typically present for several years before the manifestation of type 2 diabetes [2], is an important predictor for the development of type 2 diabetes and contributes to an elevated risk of cardiovascular disease (CVD) events [3]–[6]. Insulin secretion defect has been recognized as a key determinant for the progression to type 2 diabetes [7]–[10].

Euglycemia is maintained by a complex interaction between many tissues. In the fasting state the majority of glucose utilization takes place in the brain (∼50%), splanchnic organs (∼25%), and in skeletal muscle (∼25%) [11]. Postprandial increase in circulating insulin promotes the uptake of glucose into insulin-sensitive tissues. Glucose is stored principally in the form of glycogen mostly in the muscle (accounting for ∼60% of whole body uptake), liver (∼30%) and adipose tissue (∼10%) [11], [12]. Hepatic IR is characterized by enhanced gluconeogenesis in the fasting state and an impaired suppression of hepatic glucose production in response to insulin in the postprandial state [13]. Insulin resistance in skeletal muscle leads to postprandial hyperglycemia [14]. Adipose tissue IR results in elevated levels of plasma free fatty acids levels [15] and overproduction of proinflammatory cytokines (interleukin, tumor necrosis factor-α, C-reactive protein) which impair insulin sensitivity. Moreover, decreased insulin-mediated glucose uptake in different tissues leads to increased plasma insulin levels to maintain normoglycemia. Elevated fasting insulin concentrations reflect IR [16] and are associated with increased risk of CVD events [5], [17], [18].

Insulin sensitivity can be estimated with the hyperinsulinemic euglycemic glucose clamp [19], intravenous glucose tolerance test [20] or with indices calculated on the basis of insulin and glucose levels in the fasting state or after a glucose load [19], [21], [22]. Many of these surrogate markers have been validated against the hyperinsulinemic euglycemic glucose clamp [23]–[25]. While several tissue-specific IR indices have been developed [14], [26] it remains unknown whether they differentially predict the deterioration of hyperglycemia, progression to overt type 2 diabetes or incident CVD events, and whether they are better predictors of type 2 diabetes and CVD events than fasting insulin level. To address these questions, we investigated the ability of three validated tissue-specific IR indices to predict the deterioration of hyperglycemia, incident type 2 diabetes and CVD events in participants of the Metabolic Syndrome in Men (METSIM) Study, and compared their predictive power with that of fasting plasma insulin level.

## Materials and Methods

### Subjects and clinical measurement at the baseline study

The Metabolic Syndrome in Men (METSIM) Study was performed in 2005–2010 at the Clinical Research Unit at the University of Kuopio. It included 10,197 men, aged from 45 to 73 years, randomly selected from the population register of Kuopio, Eastern Finland (population 95,000). Each participant attended a 1-day outpatient visit to the Clinical Research Unit at the University of Kuopio, as described in detail previously [23]. An oral glucose tolerance test (OGTT, 75 g of glucose, glucose and insulin measurements at 0, 30 and 120 min) was performed, and glucose tolerance was classified according to the American Diabetes Association criteria [27]. Subjects with previously diagnosed type 1 diabetes (n = 25) or type 2 diabetes (n = 763) were excluded, and none of the participants included in statistical analyses were on anti-diabetic medication. A total of 9,398 men without diabetes or with newly diagnosed diabetes were included in the current analyses (age 57±7 years, body mass index (BMI) 27.0±4.0 kg m^−2^, mean±SD).

### Subjects and clinical measurement at the follow-up study

A follow-up started in 2010, and so far 5,419 individuals have participated. The study protocol and measurements are identical to those of the baseline study.

### Diagnosis of incident type 2 diabetes and CVD events

Out of 8,749 non-diabetic participants at baseline, 558 participants developed incident type 2 diabetes and 239 participants had a CVD event between the date of the baseline METSIM Study and 31 December 2013 (5.9-year follow-up). Diagnosis of new-onset type 2 diabetes was based either on an OGTT or HbA1c≥6.5% (297 cases of new diabetes) among 4,806 non-diabetic individuals who participated in the ongoing follow-up study to date in 2010–2013, or anti-diabetic medication started between the baseline and 31 December 2013 (n = 261 cases of new diabetes; information obtained from the National Drug Reimbursement registry for all 8,749 non-diabetic participants). The incident CVD event was defined as myocardial infarction, coronary heart disease death, or fatal and non-fatal cerebral infarction which occurred between the baseline study and 31 December 2013. CVD events were defined according to internationally accepted criteria [28], [29] and verified from the hospital records. Individuals with non-fatal myocardial infarction and stroke before the baseline were excluded from statistical analyses.

#### Ethics Statement

The study was approved by the Ethics Committee of the University of Eastern Finland and Kuopio University Hospital and was conducted in accordance with the Helsinki Declaration. All study participants gave written informed consent.

### Measurements

Height and weight were measured to the nearest 0.5 cm and 0.1 kg, respectively. Body mass index (BMI) was calculated as weight (kg) divided by height (m) squared. Waist circumference was measured at the midpoint between the lateral iliac crest and lowest rib. Body composition was determined by bioelectrical impedance (Bioimpedance Analyzer Madel BIA 101, Akern SrL, Florence, Italy) in subjects in the supine position after a 12-h overnight fast. Smoking status was defined as current smoking (yes vs. no). Family history of diabetes (yes vs. no) was defined as the first or second-degree relative having diabetes vs. no family history of diabetes. Physical activity (physically active vs. inactive) refers to leisure time exercise (physically active, regular exercise at least 30 min 1–2 times per week vs. physically inactive, occasional exercise or no exercise). Alcohol intake was defined as total alcohol intake in grams per week. Diagnosis of hypertension was based on the use of antihypertensive medication (information obtained from the National Drug Reimbursement registry).

### Laboratory measurement

Plasma glucose was measured by enzymatic hexokinase photometric assay (Konelab Systems reagents, Thermo Fisher Scientific; Vantaa, Finland). HbA1c was analyzed with a Tosoh G7 glycohemoglobin analyser (Tosoh Bioscience, Inc. San Francisco, CA, USA). Plasma insulin concentrations were measured by a luminometric immunoassay measurement (ADVIA Centaur Insulin IRI, no 02230141, Siemens Medical Solutions Diagnostics, Tarrytown, NY, USA). LDL cholesterol (LDL-C) was measured by enzymatic colorimetric test (Konelab Systems reagents, Thermo Fisher Scientific; Vantaa, Finland).

### Calculations

The trapezoidal method was used to calculate glucose area under the curve (Glucose AUC) from the OGTT using samples collected at 0, 30 and 120 min. Insulin resistance was measured by surrogate indices of tissue-specific IR. Calculation of Matsuda ISI as a measure of whole body insulin sensitivity, representing mainly skeletal muscle insulin sensitivity, has been previously described [30]. For consistency with the other indices expressed as IR, Matsuda IR (calculated as 10/Matsuda ISI) was used in all statistical analyses. The Adipocyte IR index (Adipocyte IR), was defined as the product of fasting free fatty acid levels and fasting plasma insulin [24]. We used our previously validated Liver IR index [26] as a marker of hepatic IR. Insulin secretion index (InsAUC_0–30_/GlucAUC_0–30_) was calculated as reported previously [2]. The disposition index (DI) (a measure of pancreatic β-cell function) was calculated as Matsuda ISI×InsAUC_0–30_/GlucAUC_0–30_.

### Statistical analysis

Statistical analyses were conducted using IBM SPSS Statistics version 19. All traits except for age were log-transformed to correct for their skewed distribution. IR indices and fasting plasma insulin levels were compared across the fasting plasma glucose (FPG) and 2-hour plasma glucose (2hPG) categories with ANOVA. ANCOVA was used to adjust for covariates (age and BMI). Linear regression model adjusted for the follow-up time was used to evaluate the ability of IR indices and fasting plasma insulin levels to predict worsening of FPG, 2hPG and Glucose AUC. Results are presented as unstandardized effect sizes (B and SE) and standardized regression coefficient beta. Fisheŕs r-to-z transformation was performed to test the difference between the standardized beta coefficient of IR indices and fasting insulin levels. Cox regression analysis was used to evaluate the ability of IR indices and fasting insulin levels to predict incident type 2 diabetes and CVD events. Hazard ratios (HRs) are presented with the 95% confidence intervals (CI). Linear and Cox regression models were additionally adjusted for age, BMI, smoking, physical activity, alcohol consumption, family history of type 2 diabetes, DI, Glucose AUC at baseline, and linear regression model additionally for follow-up time. Cox model evaluating the risk of incident CVD events was additionally adjusted for age, BMI smoking, physical activity, alcohol consumption, family history of type 2 diabetes, hypertension, and LDL cholesterol at baseline. *P*<0.0125 was considered as statistically significant given the 3 IR indices and fasting insulin tested. *P*<0.05 was considered as nominally significant.

## Results

### Tissue specific insulin resistance indices in the categories of glucose tolerance

**Figure 1:**
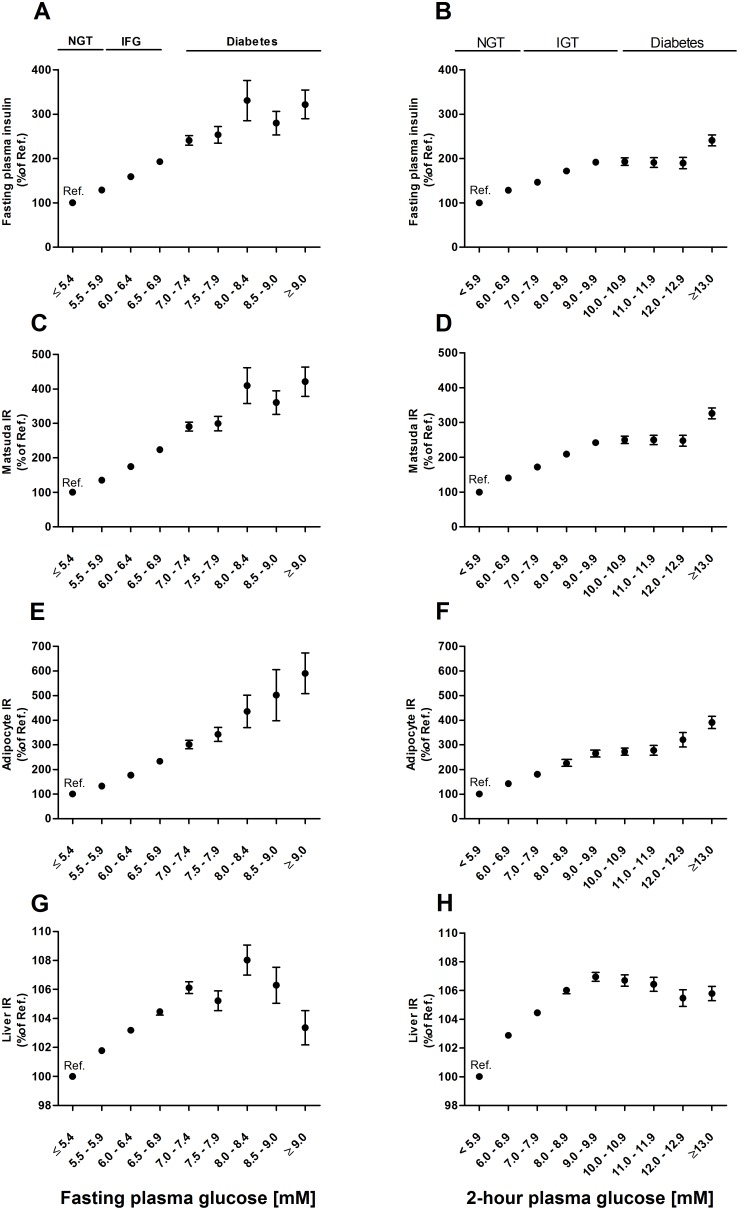
Mean values and standard errors of the mean (%) for tissue-specific insulin resistance (IR) indices across the fasting and 2-hour glucose categories. All indices and fasting insulin levels across the categories were significantly different from the reference category (fasting plasma glucose ≤5.4 mmol/L, 2-hour plasma glucose ≤5.9 mmol/L, *P*<0.001). *P* values for the trends, adjusted for age and BMI, were as follows: A) 4.7×10^−181^ B) 3.2×10^−131^ C) 6.5×10^−299^ D) <6.5×10^−299^ E) 1.7×10^−204^ F) <6.5×10^−299^ G) 1.3×10^−20^ H) 1.9×10^−119^. NGT indicates normal glucose tolerance, IFG impaired fasting glucose, and IGT impaired glucose tolerance. Participants with previously diagnosed diabetes are excluded, N = 9,398. Abbreviations: IFG, impaired fasting glucose; IGT, impaired glucose tolerance; IR, insulin resistance; NGT, normal glucose tolerance; Ref, reference.

The association of the IR indices in non-diabetic individuals and individuals with newly diagnosed type 2 diabetes across the FPG and 2hPG categories in the cross-sectional analysis of the METSIM study cohort is shown in Figure 1. Categories of FPG≤5.4 mmo/l and 2hPG≤5.9 mmol/l were set as the reference categories. The fasting plasma insulin levels increased significantly (*P* = 4.7×10^−181^ and *P* = 3.2×10^−131^, respectively) across the range of both FPG and 2hPG categories. Matsuda IR increased significantly with increasing FPG (*P* = 6.5×10^−299^) and 2hPG categories (*P*<6.5×10^−299^), adjusted for age and BMI. Compared to the reference categories, Matsuda IR increased up to +124% in the impaired fasting glucose (IFG) category and up +321% in newly diagnosed diabetes, and in the 2hPG categories up to +150% and +226% in the impaired glucose tolerance (IGT) category and in newly diagnosed diabetes respectively. Adipocyte IR also showed a significant association with the FPG (*P* = 1.7×10^−204^) and 2hPG (*P*<6.5×10^−299^) categories. Adipocyte IR increased up to +133% in individuals with IFG and up to +490% in individuals with newly diagnosed diabetes, and in the 2hPG categories up to 172% and 291% in IGT and in newly diagnosed diabetes, respectively, compared to the reference categories. Liver IR increased significantly across the entire range of FPG (*P* = 1.3×10^−20^) and 2hPG (*P* = 1.9×10^−119^). Across the FPG categories, Liver IR increased up to +4% in IFG and up to +3% in individuals with newly detected diabetes as compared to the reference category. In the 2hPG categories Liver IR increased up to +7%, and +6% in IGT and in newly diagnosed diabetes, respectively.

### Insulin resistance indices and fasting plasma insulin levels as predictors for the worsening of hyperglycemia

Matsuda IR, Adipocyte IR, Liver IR and fasting plasma insulin levels significantly predicted an increase in FPG, 2hPG and Glucose AUC (Table 1). Matsuda IR and Adipocyte IR were the best predictors of 2hPG and Glucose AUC. The associations were attenuated, but not abolished, after the adjustment for known risk factors of type 2 diabetes (age, BMI, smoking, physical activity, alcohol consumption and family history of type 2 diabetes). Additional adjustment for DI and Glucose AUC at baseline abolished the predictive value of Adipocyte IR and Liver IR in predicting FPG. Matsuda IR and Adipocyte IR were significantly better predictors for 2hPG and Glucose AUC than were fasting plasma insulin levels (Table 2).

**Table 1:** Tissue-specific insulin resistance indices and fasting plasma insulin as predictors of hyperglyceamia at the 5.9-years METSIM follow-up study, Kuopio, Finland.

	FPG at follow-up							
Index at baseline	B	SE	Beta	*P*	*P^a^*	*P^b^*	*P^c^*	*P^d^*
Matsuda IR*	0.038	0.002	0.280	**1.5E-81**	**3.4E-39**	**3.8E-13**	**5.8E-07**	**4.3E-07**
Adipocyte IR	0.028	0.002	0.248	**9.4E-64**	**6.2E-25**	0.015	**0.001**	0.217
Liver IR	0.264	0.020	0.196	**5.2E-40**	**2.1E-06**	**8.9E-08**	0.733	**2.0E-03**
Fasting plasma insulin	0.036	0.002	0.248	**4.1E-64**	**1.3E-24**	**2.9E-04**	**1.4E-08**	**6.5E-04**
**Index at baseline**	**2hPG at follow-up**							
	**B**	**SE**	**Beta**	***P***	***P^a^***	***P^b^***	***P^c^***	***P^d^***
Matsuda IR*	0.150	0.007	0.312	**1.0E-101**	**1.6E-53**	**3.0E-19**	**5.6E-07**	**4.4E-07**
Adipocyte IR	0.126	0.006	0.319	**8.1E-106**	**5.5E-59**	**6.9E-16**	**1.2E-14**	**7.0E-10**
Liver IR	1.276	0.068	0.269	**3.7E-75**	**2.1E-23**	**1.4E-28**	**2.1E-06**	**1.3E-13**
Fasting plasma insulin	0.132	0.007	0.262	**2.4E-71**	**9.5E-32**	**6.5E-06**	**1.3E-09**	**2.8E-05**
**Index at baseline**	**Glucose AUC at follow-up**							
	**B**	**SE**	**Beta**	***P***	***P^a^***	***P^b^***	***P^c^***	***P^d^***
Matsuda IR*	0.091	0.004	0.361	**2.5E-137**	**4.0E-77**	**7.9E-28**	**7.9E-04**	**7.5E-04**
Adipocyte IR	0.073	0.003	0.356	**8.0E-133**	**1.0E-73**	**2.4E-15**	**1.2E-08**	**1.2E-05**
Liver IR	0.685	0.036	0.277	**4.3E-79**	**1.2E-21**	**2.4E-29**	0.063	**9.1E-05**
Fasting plasma insulin	0.077	0.004	0.294	**5.6E-90**	**1.5E-39**	**6.6E-05**	**3.7E-07**	**4.4E-04**

Abbreviations: B, unstandardized effect size; Beta, standardized regression coefficient; FPG, fasting plasma glucose; Glucose AUC, glucose area under the curve; 2hPG, 2-hour plasma glucose; IR, insulin resistance SE, standard error.

Linear regression analysis. Values were calculated using log-transformed variables (except for age). Individuals with type 1 diabetes (N = 25), with known type 2 diabetes (N = 763), newly diagnosed type 2 diabetes at baseline (N = 649) or diagnosed with diabetes between baseline and follow-up (N = 117) were excluded, N = 4,474 for FPG and 2hPG, and 4,450 for glucose AUC. Bold font indicates statistical significance *P* ***<***0.0125.

*P*, adjusted for follow-up time.

*P* ^a^, adjusted for follow-up time, age, BMI, smoking, physical activity, alcohol consumption and family history of type 2 diabetes.

*P* ^b^, adjusted for follow-up time, age, BMI, smoking, physical activity, alcohol consumption, family history of type 2 diabetes, and disposition index at baseline.

*P* ^c^, adjusted for follow-up time age, BMI, smoking, physical activity, alcohol consumption, family history of type 2 diabetes and glucose AUC at baseline.

*P* ^d^, adjusted for follow-up time, age, BMI, smoking, physical activity, alcohol consumption, family history of type 2 diabetes, disposition index and Glucose AUC at baselin.

*Matsuda IR was calculated as: 10/Matsuda ISI.

Disposition index was calculated as: Matsuda ISI×InsAUC_0–30_/GluAUC_0–30_.

**Table 2:** Comparison of predictive ability of tissue-specific insulin resistance indices with fasting plasma insulin levels at baseline in predicting worsening of hyperglycemia at the 5.9-years METSIM follow-up study, Kuopio, Finland.

	FPG	
Indices at baseline	Comparisons of Beta coefficients	*P*
Fasting plasma insulin vs. Matsuda IR*	0.248 vs. 0.280	0.104
Fasting plasma insulin vs. Adipocyte IR	0.248 vs. 0.248	-
Fasting plasma insulin vs. Liver IR	**0.248 vs.0.196**	**0.010**
	**2hPG**	
**Indices at baseline**	**Comparisons of Beta coefficients**	***P***
Fasting plasma insulin vs. Matsuda IR*	**0.262 vs. 0.312**	**0.010**
Fasting plasma insulin vs. Adipocyte IR	**0.262 vs. 0.319**	**0.003**
Fasting plasma insulin vs. Liver IR	0.262 vs. 0.269	0.722
	**Glucose AUC**	
**Indices at baseline**	**Comparisons of Beta coefficients**	***P***
Fasting plasma insulin vs. Matsuda IR*	**0.294 vs. 0.361**	**<0.001**
Fasting plasma insulin vs. Adipocyte IR	**0.294 vs. 0.356**	**<0.001**
Fasting plasma insulin vs. Liver IR	0.294 vs. 0.277	0.383

Abbreviations: Beta, standardized regression coefficient; FPG, fasting plasma glucose; Glucose AUC, glucose area under the curve; 2hPG, 2-hour plasma glucose; IR, insulin resistance.

Beta was obtained from linear regression analysis. The difference between the standardized coefficients of IR indices and fasting insulin was tested using Fisheŕs r-to-z transformation. Individuals with type 1 diabetes (N = 25), with known type 2 diabetes (N = 763), newly diagnosed type 2 diabetes at baseline (N = 649) or diagnosed with diabetes between baseline and follow-up (N = 117) were excluded, N = 4,474 for FPG and 2hPG, and 4,450 for glucose AUC. Bold font indicates statistical significance *P*<0.0125. *P*, unadjusted.

*Matsuda IR was calculated as: 10/Matsuda ISI.

### Insulin resistance indices and fasting plasma insulin levels as predictors of incident diabetes and CVD events

A total of 558 of 8,749 non-diabetic men at baseline developed incident type 2 diabetes and 239 men a CVD event. In unadjusted Cox regression models all tissue-specific IR indices and fasting plasma insulin levels significantly predicted an increased risk of incident type 2 diabetes (Table 3) and incident CVD events (Table 4). Liver IR was the strongest predictor of both incident type 2 diabetes (HR = 1.83, 95% CI: 1.68–1.98, *P* = 3.80×10^−46^) and CVD events (HR = 1.31, 95% CI: 1.15–1.48, *P* = 4.0×10^−5^). Matsuda IR, Adipocyte IR and fasting plasma insulin levels had comparable ability to predict incident type 2 diabetes and CVD events.

**Table 3:** Tissue-specific insulin resistance indices and fasting plasma insulin as predictors of incident type 2 diabetes in the 5.9-years METSIM prospective study, Kuopio, Finland.

	Incident type 2 diabetes								
	N								
Index at baseline	Events	Total	HR	95% CI	*P*	*P^a^*	*P^b^*	*P^c^*	*P^d^*
Matsuda IR*	556	8,697	1.46	1.40–1.52	**1.3E-70**	**2.2E-19**	**0.013**	0.746	0.985
Adipocyte IR	557	8,743	1.36	1.31–1.42	**1.3E-48**	**7.0E-11**	0.950	0.435	0.092
Liver IR	555	8,674	1.83	1.68–1.98	**3.8E-46**	**1.4E-08**	**1.7E-04**	0.855	0.237
Fasting plasma insulin	557	8,745	1.37	1.32–1.42	**2.3E-59**	**1.1E-10**	0.347	0.073	0.600

Abbreviations: CI, confidence interval; HR, hazard ratio; IR, insulin resistance.

HRs and their 95% CI were obtained from Cox regression analyses. HRs were calculated using standardized predictors (unit = one standard deviation). Individuals with type 1 diabetes (N = 25), type 2 diabetes (N = 763) or newly diagnosed type 2 diabetes at baseline (N = 649) were excluded. A total of 558 individuals were diagnosed with incident type 2 diabetes during the 5.9 year follow-up, 8,191 participants remained non-diabetic. Bold font indicates statistical significance *P<*0.0125.

*P*, unadjusted.

*P* ^a^, adjusted for age, BMI, smoking, physical activity, alcohol consumption and family history of type 2 diabetes.

*P^b^*, adjusted for age, BMI, smoking, physical activity, alcohol consumption, family history of type 2 diabetes and disposition index at baseline.

*P* ^c^, adjusted for age, BMI, smoking, physical activity, alcohol consumption, family history of type 2 diabetes and glucose AUC at baseline.

*P* ^d^ adjusted for age, BMI, smoking, physical activity, alcohol consumption, family history of type 2 diabetes, disposition index and glucose AUC at baseline.

*Matsuda IR was calculated as: 10/Matsuda ISI.

Disposition index was calculated as: Matsuda ISI×InsAUC_0–30_/GluAUC_0–30_.

**Table 4:** Tissue-specific insulin resistance indices and fasting plasma insulin as predictors of total incident CVD events in the 5.9-Years METSIM prospective study, Kuopio, Finland.

	Incident CVD events					
	N					
Index at baseline	Event	Total	HR	95%CI	*P*	*P^a^*
Matsuda IR*	237	8,301	1.14	1.02–1.27	0.021	0.559
Adipocyte IR	239	8,346	1.12	1.01–1.24	0.032	0.536
Liver IR	237	8,283	1.31	1.15–1.48	**4.0E-05**	0.126
Fasting plasma insulin	239	8,348	1.11	1.00–1.24	0.049	0.599

Abbreviations: CI, confidence interval; CVD, cardiovascular disease; HR, hazard ratio; IR, insulin resistance; LDL-C low-density lipoprotein cholesterol.

Cox regression analysis. HRs were calculated using standardized predictors (unit = one standard deviation). Individuals with previously or newly diagnosed type 2 diabetes and type 1 diabetes at baseline were excluded from the analyses, as well as individuals with myocardial infarction and stroke before the baseline. Bold font indicates statistical significance *P*<0.0125. Individuals with type 1 diabetes (N = 25), type 2 diabetes (N = 763) or newly diagnosed type 2 diabetes at baseline (N = 649) were excluded.

*P*, unadjusted.

*P* ^a^, adjusted for age, BMI smoking, physical activity, alcohol consumption and family history of type 2 diabetes, hypertension (diagnosis was based on drug reimbursement) and LDL-C at baseline.

*Matsuda IR was calculated as: 10/Matsuda ISI.

## Discussion

Insulin resistance is an important predictor for the development of type 2 diabetes and contributes to an elevated risk of CVD events [3]–[5], [7]–[9], [17]. None of the previous studies has investigated the association of different markers of tissue level IR with the worsening of hyperglycemia, conversion to type 2 diabetes or incident CVD events. Our study showed that Matsuda IR (representing mainly IR in the skeletal muscle), Adipocyte IR (representing IR in the adipose tissue), Liver IR (representing IR in the liver) and fasting plasma insulin levels significantly predicted the worsening of hyperglycemia and the conversion to type 2 diabetes, independently of the confounding factors (age, BMI, smoking, physical activity, alcohol consumption, family history of diabetes, insulin secretion and baseline glycemia). Importantly, Matsuda IR and Adipocyte IR were superior to fasting insulin levels in predicting 2hPG and Glucose AUC. All markers of IR significantly predicted an increased risk of CVD events in unadjusted models.

Hyperinsulinemic euglycemic clamp is the gold standard for measuring whole-body insulin sensitivity [11]. Combined with radiolabelled glucose it allows to quantify individual contribution of liver and muscle insulin resistance to impaired whole-body glucose disposal [30]. These techniques are not, however, applicable to large population-based studies. Therefore, markers of IR suitable for large-scale studies have been developed. An index of muscle insulin sensitivity developed by Abdul-Ghani et al. [4] is based on the measurement of glucose and insulin during an OGTT at 8 time points. This index had a correlation of 0.78 with the M value from the clamp [4]. Because several blood samples are needed, this measure is not possible to apply in large-scale population-based studies. Instead, we calculated Matsuda IR based on three measurements (0, 30 120 min) during an OGTT. We have previously shown that Matsuda ISI, based on five measurements (0, 30, 50, 90 and 120 min), has a correlation of 0.77 with the M value from the clamp, and that Matsuda ISI based on five and three measurements gives very similar results (correlation of 0.985) [31]. Therefore, we believe that 10/Matsuda ISI reliably measures skeletal muscle IR in insulin-stimulated state. We have developed a new index of Liver IR having a correlation of 0.65 with a product of tracer measured hepatic glucose production and fasting insulin [26]. This index was used as a surrogate marker of liver IR in our statistical analyses. Insulin resistance in adipose tissue leads to an impairment of insulin’s antilipolytic effect, contributing to increased flux of free fatty acids into the circulation. We estimated Adipocyte IR with an index validated previously, calculated as the product of the fasting plasma insulin concentration and the fasting plasma FFA concentration [32].

Matsuda IR, reflecting insulin resistance mainly in the skeletal muscle, and Adipocyte IR had comparable ability to predict the worsening of hyperglycemia, measured by 2hPG and Glucose AUC in an OGTT, after adjustment for all confounding risk factors, including insulin secretion and baseline Glucose AUC. This is not surprising since skeletal muscle is responsible for >60% of glucose disposal in insulin-stimulated states. Both indices predicted better an increase in 2hPG and Glucose AUC at follow-up than fasting plasma insulin levels. Liver IR was the strongest predictor of the conversion to type 2 diabetes (HR = 1.83, 95% CI: 1.68–1.98, *P* = 3.80×10^−46^) and incident CVD events (HR = 1.31, 95% CI: 1.15–1.48, *P* = 4.0×10^−05^) in our 5.9-year follow-up study, although Matsuda IR, Adipocyte IR and fasting plasma insulin levels also significantly predicted incident type 2 diabetes and CVD events.

The strengths of our study include the examination of a large, population-based sample of men across a broad age range and standardized assessment of diabetes with an OGTT, and the measurement of tissue-level IR with validated markers. Limitations of our study are that it included only men, and one ethnic group. Although we used validated markers for IR, direct physiological measurement of insulin sensitivity at tissue level (euglycemic clamp, radiolabelled glucose etc.) are more accurate. However, direct tissue level measurements are not possible to perform in studies of several thousand participants.

In conclusion, tissue-specific markers of IR predicted the worsening of hyperglycemia, incident type 2 diabetes and CVD events, independently of confounding risk factors. Matsuda IR and Adipocyte IR were stronger predictors of the worsening of 2hPG and Glucose AUC than fasting plasma insulin levels. Liver IR was the best predictor for incident type 2 diabetes and CVD events. Our study shows that tissue-specific markers of IR offer a new approach to evaluate the role of insulin resistance beyond and above fasting insulin level in the prediction of incident type 2 diabetes and cardiovascular disease.
