# Correction: Markers of Tissue-Specific Insulin Resistance Predict the Worsening of Hyperglycemia, Incident Type 2 Diabetes and Cardiovascular Disease

**DOI:** 10.1371/journal.pone.0114705

**Published:** 2014-12-05

**Authors:** The PLOS ONE Staff

The first author's name is spelled incorrectly. The correct name is: Mária Fízeľová. The correct citation is: Fízeľová M, Cederberg H, Stančáková A, Jauhiainen R, Vangipurapu J, et al. (2014) Markers of Tissue-Specific Insulin Resistance Predict the Worsening of Hyperglycemia, Incident Type 2 Diabetes and Cardiovascular Disease. PLoS ONE 9(10): e109772. doi:10.1371/journal.pone.0109772
